# A New and Fast Technique to Generate Offspring after Germ Cells Transplantation in Adult Fish: The Nile Tilapia (*Oreochromis niloticus*) Model

**DOI:** 10.1371/journal.pone.0010740

**Published:** 2010-05-20

**Authors:** Samyra M. S. N. Lacerda, Sergio R. Batlouni, Guilherme M. J. Costa, Tânia M. Segatelli, Bruno R. Quirino, Bruno M. Queiroz, Evanguedes Kalapothakis, Luiz R. França

**Affiliations:** 1 Laboratory of Cellular Biology, Department of Morphology, Federal University of Minas Gerais, Belo Horizonte, Minas Gerais, Brazil; 2 São Paulo State University, Aquaculture Center (CAUNESP), Jaboticabal, São Paulo, Brazil; 3 GM Alevinos LTDA, Contagem, Minas Gerais, Brazil; 4 Laboratory of Biotechnology and Molecular Markers, Department of General Biology, Federal University of Minas Gerais, Belo Horizonte, Minas Gerais, Brazil; Institute of Zoology, Chinese Academy of Sciences, China

## Abstract

**Background:**

Germ cell transplantation results in fertile recipients and is the only available approach to functionally investigate the spermatogonial stem cell biology in mammals and probably in other vertebrates. In the current study, we describe a novel non-surgical methodology for efficient spermatogonial transplantation into the testes of adult tilapia (*O. niloticus*), in which endogenous spermatogenesis had been depleted with the cytostatic drug busulfan.

**Methodology/Principal Findings:**

Using two different tilapia strains, the production of fertile spermatozoa with donor characteristics was demonstrated in adult recipient, which also sired progeny with the donor genotype. Also, after cryopreservation tilapia spermatogonial cells were able to differentiate to spermatozoa in the testes of recipient fishes. These findings indicate that injecting germ cells directly into adult testis facilitates and enable fast generation of donor spermatogenesis and offspring compared to previously described methods.

**Conclusion:**

Therefore, a new suitable methodology for biotechnological investigations in aquaculture was established, with a high potential to improve the production of commercially valuable fish, generate transgenic animals and preserve endangered fish species.

## Introduction

Spermatogonial stem cell transplantation is a fascinating and very promising reproductive technology developed in 1994 by Brinster and collaborators [1]. Although clearly presenting phylogenetic limitations for different mammalian species [2], the transplantation of spermatogonial cells between males can result in a recipient animal producing fertile spermatozoa that carry the donor genotype. First established in rodents, spermatogonial stem cell transplantation has now been used in other mammalian species and enabled tremendous progress investigating the phenotypic and functional characteristics of this fundamental testicular stem cell. This technique also represents a valuable tool for studies involving biotechnology, *in vitro* culture, cryopreservation, transgenic animal production and the preservation of genetic stocks of valuable animals or endangered species [3]. In fish, germ cell transplantation has been performed using primordial germ cells (PGCs) [4] or spermatogonia [5], [6] microinjected into the coelomic cavity of newly hatched salmon and trout embryos, migrating thereafter to the undifferentiated gonads in a narrow window of development determined by the migration of the endogenous primordial germ cells. However, one substantial limitation to this technique is that it takes more than a year for the recipient salmonid gonads to become functionally mature and produce fertile sperm [4], [5], [6]. More recently, using a surgical procedure, it has been suggested that germ cell transplantation in juvenile fish could potentially be used as an approach to preserve endangered fish species [7].

In recent years, the Nile tilapia (*Oreochromis niloticus*; Cichlidae) has become the farmed fish species with the largest production expansion in aquaculture worldwide [8]. In addition to its fast growth rate, early sexual maturation and its capacity to adapt to a wide range of environmental and management conditions, the Nile tilapia has a great potential for experimental investigations, particularly those related to reproductive biology [9]. Different from mammals, in teleost fish spermatogenesis occurs synchronously within cysts and is maintained by self-renewal and differentiation of the spermatogonial stem cells to produce spermatozoa throughout the lifetime of animals [10]. Therefore, studying spermatogonial stem cell biology can provide unique opportunities to improve our understanding of fish spermatogenesis and to develop biotechnologies applied to these vertebrates. In this regard, we have recently established in our laboratory all the necessary procedures for intraspecific/syngeneic spermatogonial stem cell transplantation in sexually mature tilapia, such as for instance the depletion of endogenous spermatogenesis using busulfan in association with a high temperature (35°C), isolation of tilapia spermatogonial cells and labeling of donors germ cells [11].

The present investigation reports the fast generation of donor-derived spermatozoa and production of normal offspring of donor origin. Moreover, we demonstrate the functionality of cryopreserved tilapia spermatogonial cell that, after being transplanted, effectively colonized and were able to differentiate into spermatozoa in recipient testis.

## Materials and Methods

### Ethics Statement

Animal handling and experimentation were consistent with Brazilian national regulation and were approved by the Ethics Committee on Animal Care of the Federal University of Minas Gerais (CETEA - # 071/05).

### Donor and recipient animals

For experiments to investigate the development of injected fresh and frozen/thawed spermatogonia and generation of spermatozoa after transplantation, wild-type coloration adult Nile tilapia (Chitralada strain) were used as donors and recipients fishes. For experiments to evaluate the functionality/fertility of donor-derived spermatozoa produced after transplantation, adult hybrid Red tilapia and adult Chitralada tilapia were used as donors and recipient fishes respectively.

### Recipient preparation

To deplete the endogenous spermatogenesis of recipient tilapia, the fishes (n = 50) were kept at temperatures of 35°C for at least two weeks before receiving intraperitoneally two busulfan (Sigma, St. Louis, MO, USA) injections (18 mg/kg body weight and 15 mg/kg body weight), with a two-week interval between injections [11].

### Donor cell isolation

Germ cells were harvest from the testes of adult males (Red and Chitralada tilapia) through enzymatic digestion [11], [12]. Briefly, testes were dissociated with 2% collagenase (Sigma, St. Louis, MO) in Dulbecco Modified Eagle medium/Ham F-12 medium (DMEM/F12- Gibco, Grand Island, NY) for four hours at 25°C. The dispersed testicular tissue was then incubated with 0.25% trypsin/1mM EDTA and 0.03% DNase I for 30 minutes under similar conditions. An equal volume of fetal bovine serum (FBS, Gibco) was used to inactivate the trypsin. The cell suspension was filtered through a 60 µm mesh, centrifuged at 200×g for 10 minutes and re-suspended in DMEM/F12. An enriched type A spermatogonia cell suspension was obtained by percoll gradient centrifugation according to methods previously described [11], [13]. After enrichment, the cell suspension was pooled for differential plating to remove eventual testicular somatic cells [14], [15]. A total of 1.5×10^7^ cells per dish (60cm^2^, TPP, Switzerland) were cultured in DMEM/F12 supplemented with 10% FBS, 10000U/L penicillin, 10mg/L streptomycin and 10mM Na_2_HCO_3_ (Sigma) for 12 hours at 25°C in an atmosphere of 5% CO_2_. Since testicular somatic cells are able to attach to the culture dish this procedure allowed satisfactory purification of germ cell.

### Donor germ cell labeling and transplantation

Before transplantation into the testes of sexually mature tilapia, germ cells were incubated with the fluorescent membrane dye PKH26-GL (Sigma, St. Louis, MO), which is easily traced to identify the transplanted cells in the recipient testes [16]. The staining was performed following the manufacture's guidelines with the optimal final concentration of 9µM PKH26. In adult tilapia, the only non-surgical access to the seminiferous tubules is via the common spermatic duct that opens in the urogenital papilla through the urogenital pore. Thus, three weeks after the first busulfan injection, recipient tilapia were anesthetized with Quinaldin solution (1∶5000 in water; Merck & Co.) and then received the donor germ cells through the common spermatic duct using a glass micropipette (outside diameter 70 µm) under a stereomicroscope (Olympus SZX-ILLB2-100). The collected cells were suspended in DMEM/F12 and 0.4% trypan blue solution (1∶10) at a concentration of 10^7^ cells/mL. The injected volume was approximately 1 mL for each recipient. As tilapia usually reproduce in the temperature of 24°C to 26°C, after the transplant the water temperature was gradually decreased (1–2°C per day) from 35°C to 25°C.

### Microscopic observation of donor-derived germ cells in recipient tilapia

To evaluate the establishment or efficiency of the colonization of fresh and frozen donor germ cells post-transplantation and the development (proliferation, differentiation) of these cells in the recipient gonads, the testes that received PKH26-labeled germ cells were collected at specific time periods (from 1 hour to 11 weeks), immediately embedded in Jung Tissue Freezing Medium (Leica Instruments, Nussloch, Alemanha), frozen in liquid nitrogen and stored at −80°C. Testis samples were cryosectioned serially and stained with DAPI (targeting DNA in the cell nucleus). The obtained sections were analyzed under fluorescent (Olympus IX-70) and confocal microscopies (LSM 510 Meta Zeiss, Oberkochen, Germany). Testis fragments were also routinely prepared for conventional light microscopy investigations.

### Parentage analysis

One single experiment was developed for this purpose. Total genomic DNA of donors (red tilapia; n = 15), recipients (Chitralada tilapia; n = 4); females (Chitralada tilapia, n = 4) and F1 individuals fries with approximately two weeks of age post-fertilization (n = 32) was extracted from dorsal fins or muscles using the Chelex/proteinase-K technique. For each PCR reaction, 75 ng of tilapia genomic DNA, 5× IVB buffer, 40 µM dNTPs, 5 pmol forward primer, 5 pmol reverse primer and 1 unit of *Taq* DNA polymerase (Phoneutria, Brazil) were used in a total volume of 25 µl. Tilapia DNA microsatellite marker was used to evaluate the genetic identity of the fish: UNH 104- GenBank G12257 [17]. Thermal cycling was performed using an MJ Research PTC-100. After initial denaturation for 3 min at 95°C, DNA was amplified in 5 cycles of polymerase chain reaction (30s at 95°C, 35s at 50°C and 30s at 72°C) followed by another 25 cycles (30s at 95°C, 35 s at 48°C and 30s at 72°C) and completed with a final elongation step of 4 min at 72°C. Nine microliters of each reaction were loaded onto 6% polyacrilamyde gel and electrophoresis was carried out using 1× TBE buffer. The molecular weight of the DNA fragments was estimated using a 25-pb ladder marker (Invitrogen) and the samples were analyzed using the Diversity Database software (Bio-Rad).

### Cryopreservation of tilapia germ cells

Testes from sexually mature tilapia (n = 20) were digested and a spermatogonium-enriched cell suspension was obtained as described above. Cryopreservation was performed using slightly modified methods described by Avarbock and colleagues [18]. Briefly, aliquots of 500 µL of cell suspension (10^5^ cells/mL) were carefully added to an equal of volume of freezing medium (10% fetal bovine serum, 80% DMEM/F12, 10% DMSO - Sigma, St. Louis, MO) and distributed in 1.5 mL freezing vials. Samples were placed in an ultrafreezer at −80°C and, after 12 h, were transferred to liquid nitrogen (−196°C). For thawing three weeks after cryopreservation, the cryotubes were placed in a water bath for 1 to 2 min at 25°C and the cryoprotective agent was removed. The trypan blue exclusion test was used to evaluate cell viability. The proliferative activity/viability of thawed spermatogonial cells (DNA synthesis) was assessed using tritiated thymidine (1 µCi/mL) incorporation into the culture for 48 hours. The cells were then pelleted, fixed with 4% buffered glutaraldehyde and routinely prepared to detect the thymidine labeling. Thawed spermatogonial cells were transplanted to sexually mature tilapia (n = 8) as described above.

## Results

### Transplantation of donor spermatogonia into busulfan-treated tilapia

**Figure 1:**
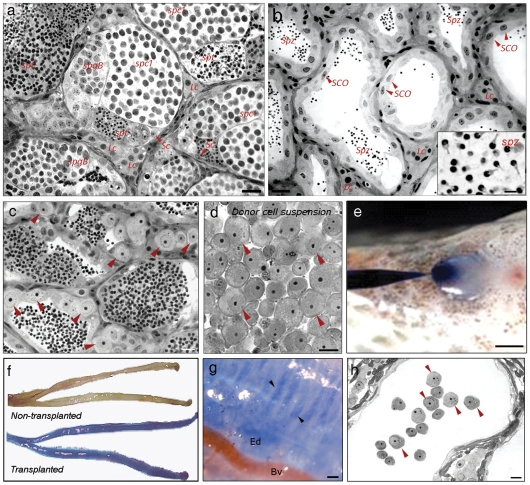
Preparation and injection of donor germ cell suspension into the adult recipient tilapia testis. Histology of sexually mature tilapia testis kept at 35°C, control (a) and treated with busulfan (b). Observe that seminiferous tubules of control fish show spermatogenic cysts in different phases of development (a) whereas Sertoli cells-only (*SCO*) are present in most of seminiferous tubule of busulfan-treated tilapia, though old spermatozoa (*spz*) produced before or during busulfan-treatment is frequently found in the seminiferous tubule lumen (b). (c) Adult tilapia testis showing the typical morphology of type A spermatogonia (arrow heads). (d) Donor germ cell suspension enriched with type A spermatogonia (arrow heads) obtained by density gradient centrifugation (percoll) and differential plating. For donor germ cell injection, a micropipette is inserted into the common spermatic duct that opens in the urogenital papillae (e). After injection, the transplanted testes are stained blue (f) due to the presence of trypan blue in the injected cell suspension. The appearance of the testes in a recipient tilapia, which did not receive germ cell transplantation, is also shown in the upper part of Figure 1f. At a higher magnification, in transplanted testis, the seminiferous tubule lumina are completely filled with the injected blue solution (g, arrow heads). One hour after transplantation, donor spermatogonia are observed in the lumen of a recipient seminiferous tubules (h, arrow heads). *spgB*: type B or secondary spermatogonia; *spc1*: primary spermatocytes; *spt*: spermatids; Lc: Leydig cell; Ed: Efferent ductules region; Bv: Blood vessels. Scale bar: a–b = 20µm; insert in b = 4µm; c, d, h = 10µm; e = 3mm; g = 70µm.

The depletion of endogenous spermatogenesis, following busulfan treatment in association with the temperature of 35°C, was verified in recipient testes of tilapia sacrificed at the time of transplantation. Different from control testis (Fig. 1a), treated tilapia rarely presented endogenous spermatogenic cysts three weeks after the first busulfan injection (Fig. 1b). In the current study, donor spermatogonial cells were obtained from adult tilapia testes. Histological studies described two subtypes of type A undifferentiated spermatogonia (presumably stem cells) in the testis of Nile tilapia [10] and both are large single cells presenting a prominent nucleolus (*spgA*, Fig. 1c). As in fish in general and in tilapia in particular specific molecular markers for spermatogonial stem cells are not yet known [10], the type A spermatogonial population obtained to be transplanted in the present study was identified and selected according to their size and morphologic characteristics (Fig. 1d). Thus, an enriched type A spermatogonial cell suspension was transplanted into the testes in a single non-surgical procedure through the spermatic duct (Fig. 1e) of recipient adult tilapia, in which endogenous spermatogenesis had been depleted with busulfan. To monitor the injection efficiency, a trypan blue solution was added to the germ cell suspension. Following transplantation, the recipient testes were stained completely blue (Fig. 1f), indicating that the cell suspension had access to all seminiferous tubules (Fig. 1g). Within one to two hours after transplantation, microscopic analysis of recipient testes demonstrated that donor spermatogonia were present in the lumen of seminiferous tubules (Fig. 1h).

**Figure 2:**
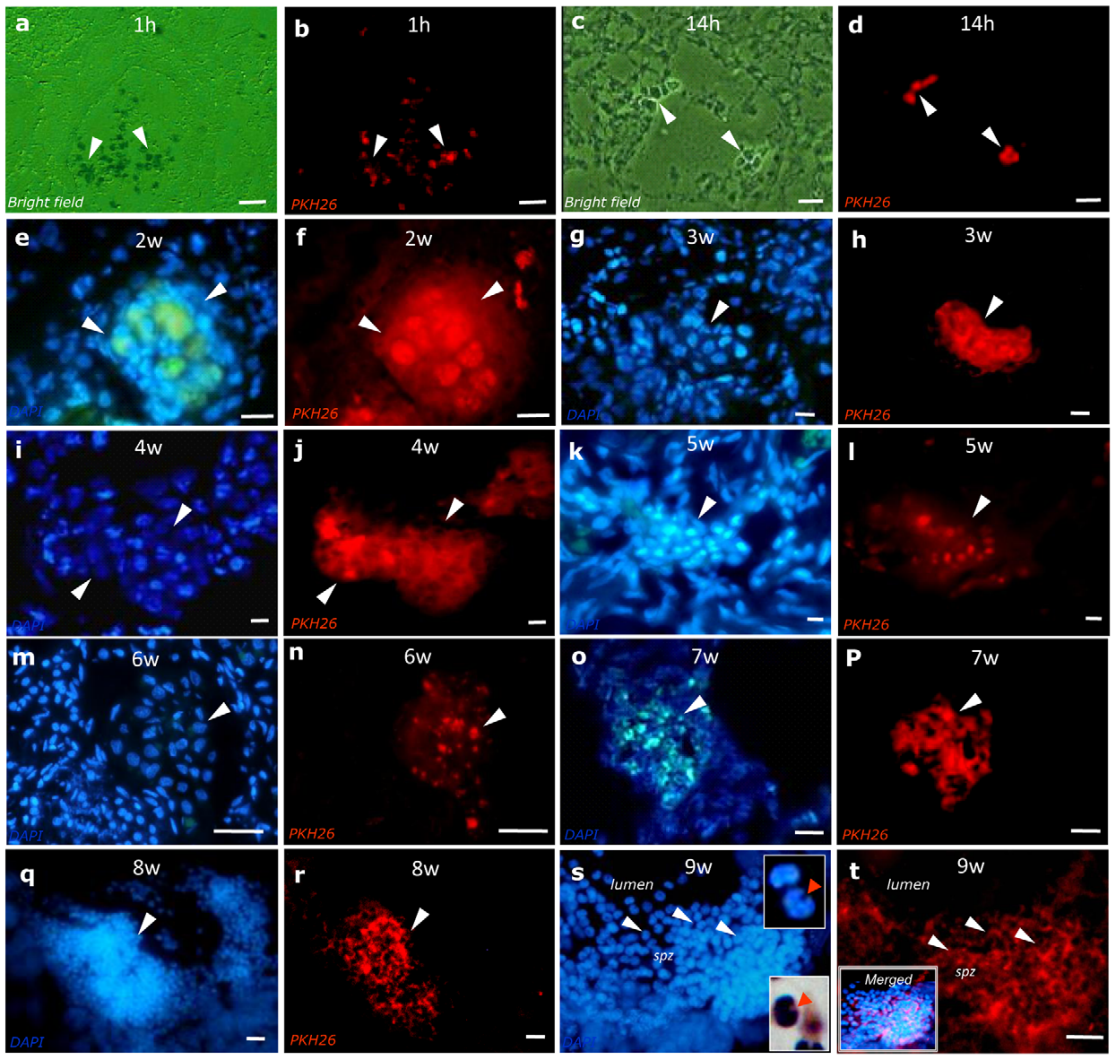
Microscopic evaluation of recipient tilapia testis following syngeneic spermatogonial transplantation. The post-transplantation interval (in hours/weeks) is shown at the top of each figure panel. Isolated PKH26 labeled germ cells (in red) were observed in the seminiferous tubules lumina (b; arrow heads) or resting on the tubule walls (d; arrow heads), respectively, at one and fourteen hours post-transplantation. The same areas shown in the panels “b” and “d” represent testis sections under bright field microscopy. From two to eight weeks after transplantation (f, h, j, l, n, p, r; arrow heads), these PKH26 labeled germ cells, also labeled with DAPI (in blue; e, g, i, k, m, o, q), established evident spermatogenic cysts of different sizes. Nine weeks after transplantation, donor-derived spermatozoa (*spz*) labeled with DAPI (s; arrow heads) and also labeled with PKH26 (t; arrow heads) were observed in the recipient seminiferous tubule lumen. At higher magnification, inserts in “s” show the sperm flagellum implantation fossa (top insert; arrow head), also illustrated by light microscopy (bottom insert; arrow head); whereas the insert in “t” represents the merged images from “s” and “t”. Scale bars: a–b = 50µm; c–t = 10µm.

The transplantation of genetically marked germ cells is a good approach for following the donor cell fate in recipient seminiferous tubules [5], [19]. As models carrying a reporter transgene expressed in male germ cells, such as *lacZ* or *gfp*, are not yet available for tilapia, a red fluorescent cell linker (PKH26-GL, a lipophilic cell membrane dye) was used to label and track donor-derived germ cells after transplantation [20], [21]. The analysis of recipient testes by fluorescence microscopy at 1 and 14 h post-transplantation revealed the presence of donor germ cells in the lumen (Fig. 2a–b) and in contact with recipient Sertoli cells (Fig. 2c–d). PKH26 labeled germ cells in a typical cystic arrangement were evident after the second week following transplantation. These spermatogenic cysts were found in different sizes and presumably at different stages of development of spermatogenesis (Fig. 2e–p). At approximately eight to nine weeks following transplantation, spermatids and spermatozoa labeled with PKH26 and arranged in cystic structures and in the lumen of seminiferous tubules, respectively, were found in the recipient testes (Fig. 2q–t). Overall, fluorescent-labeled donor germ cells in different phases of development were identified in multiple seminiferous tubules in 89% (34/38) of recipient testes (Table S1). Suggesting that donor spermatogonia can self-renew and/or stay longer in the testes, isolated PKH26 labeled spermatogonia surrounded by somatic cells were still observed in the seminiferous epithelium several weeks post-transplantation (Figure S1). It is worth mentioning that, due to the procedures of freezing and cryosectioning used most of the times when evaluating the recipient tilapia testes fragments, the shape of the germ cells present inside the spermatogenic cysts may be distorted.

**Figure 3:**
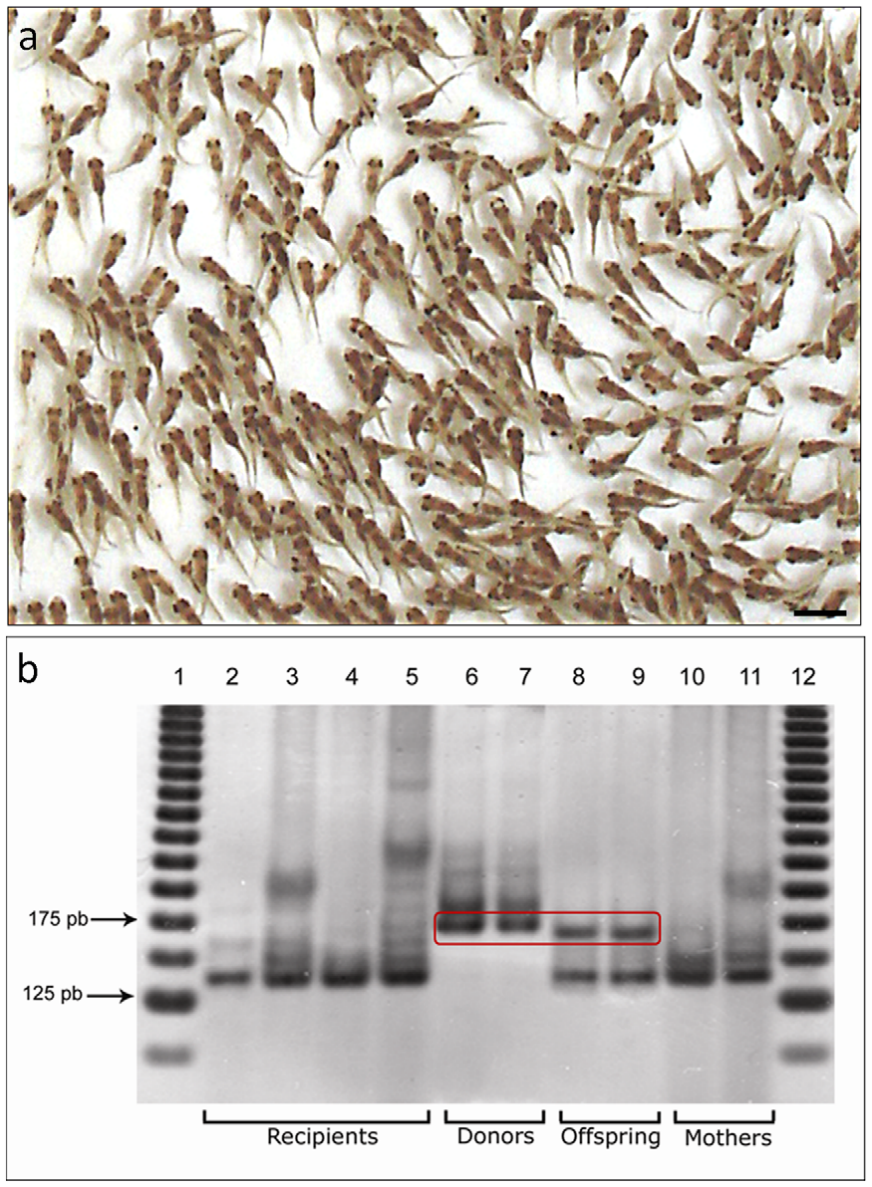
Offspring and parentage analysis. (a) Normal offspring generate from the cross between transplanted male and female chitralada tilapias. Scale bar = 0.9cm (b) Genomic DNA analysis of *O. niloticus* microsatellite marker UNH104. Genotype of recipient tilapia (lanes 2–5), donors (lanes 6 and 7), offspring (lanes 8 and 9) and mothers (lanes 10 and 11) are shown. Alleles from donors (171pb) were detected in two progeny individuals (rectangle), indicating that these fish were derived from donor cells and were not related to the surrogate recipients. Lanes 1 and 12 show the internal size standards (DNA ladder).

The breeding of the red tilapia (*O. niloticus*) with Chitralada tilapia (*O. niloticus*) resulted in a ratio of approximately 2∶1∶1 skin pigmentation phenotype of Chitralada tilapia, spotted and red tilapia, respectively (Figure S2). Therefore, to investigate the functionality of the donor-derived sperm produced by spermatogonial transplantation, spermatogonia isolated from red tilapia were transplanted into the testes of busulfan-treated wild-type coloration Nile tilapia (Chitralada strain). Nine weeks post-transplantation, the recipient fishes were used as broodstock with females from the same strain (Chitralada). The crosses generated normal offspring (Fig. 3a). Because at the early stage of development the fries do not present differentiate skin color pattern [22], [23] to clearly distinguish the individuals based on their color phenotype [24], a parentage analysis was performed on thirty-two larvae produced from these crossbreeds using microsatellite marker (UNH104) to determine their genetic identity. DNA microsatellite analysis revealed the presence of donor alleles in two individuals (6.3%) of the F1 progeny (Fig. 3b). These data demonstrated that the donor spermatogonial cells were the source of the genetic material used to produce the sperm that generated descendants of the recipient male's offspring. Donor-derived germ cells from syngeneic transplantation were demonstrated to differentiate into fully functional and fertile spermatozoa in the recipient testes.

**Figure 4:**
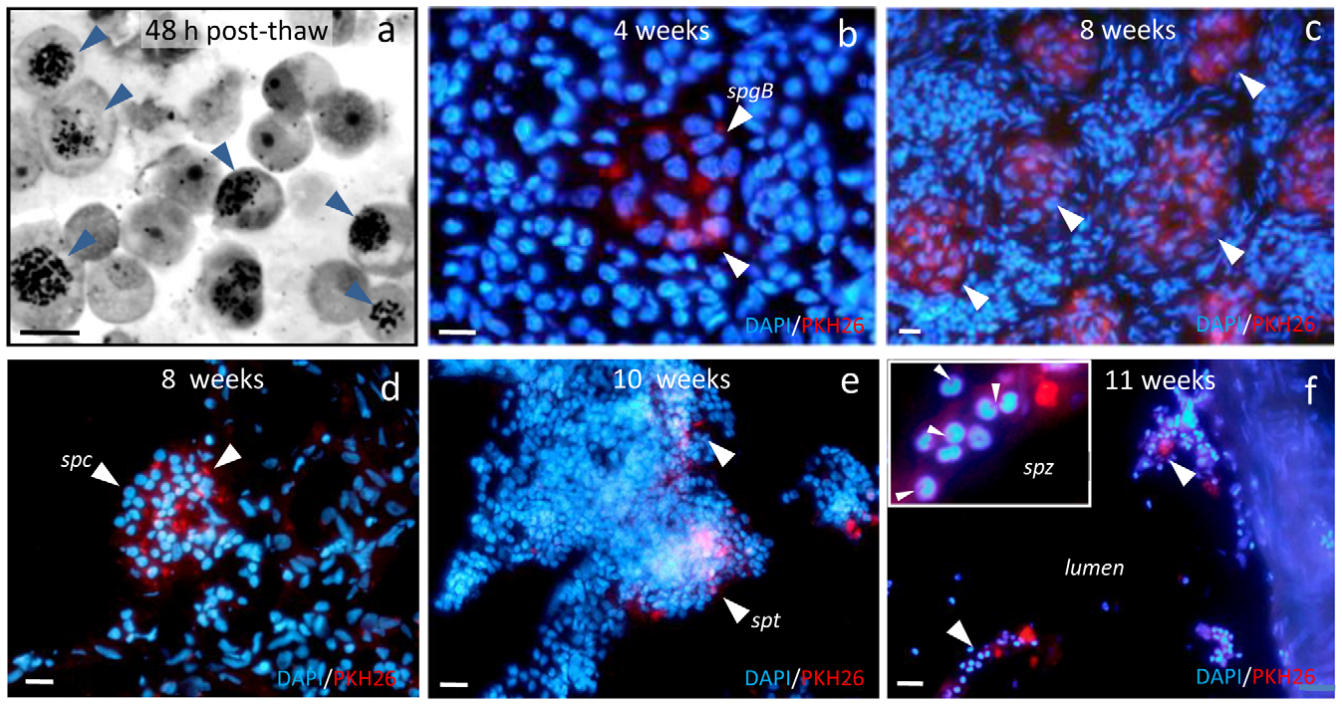
Cryopreserved spermatogonial cell transplantation. (a) Spermatogonial cells labeled with tritiated thymidine showing proliferative activity (arrow heads) after frozen/thawing. Four (b), eight (c, d) and ten weeks (e) post-transplantation, donor labeled germ cells (in red) in cystic arrangement were observed in the recipient seminiferous tubules (arrow heads). Based on the number and size of PHK26 labeled germ cells, cysts with appearance of type B spermatogonia (*spgB*), spermatocyts (*spc*) and spermatids (*spt*) were found. Eleven weeks after transplantation, PKH26 labeled donor-derived spermatozoa (*spz*) were detected in the recipient seminiferous tubule lumen (f, arrow heads). Insert in “f” shows the spermatozoa flagellum implantation fossa at higher magnification (arrow heads). Panels “b” to “f” show merged fluorescence images. The nuclei are identified by DAPI staining (blue). Scale bars: a–f = 10 µm; insert = 2 µm.

To investigate whether tilapia spermatogonia could be cryopreserved and, after thawing, generate normal spermatogenesis following transplantation to a recipient, germ cells suspensions from adult fish were subjected to the standard procedure used for freezing mouse spermatogonial stem cells [18]. Frozen/thawed spermatogonia survived in culture and retained the ability to proliferate as determined by tritiated thymidine incorporation assays (Fig. 4a). To test the functionality of tilapia spermatogonial cells after cryopreservation, frozen/thawed cells were transplanted into adult testes. As evidenced by fluorescence microscopy, the donor germ cells efficiently colonized the testes and generated PKH26-labeled spermatogenic cysts (Fig. 4b–d) in 88% of recipient fish (7/8; Table S2) and ultimately formed mature spermatids (Fig. 4e) and spermatozoa (Fig. 4f) ten to eleven weeks post-transplantation.

## Discussion

This is the first report on successful, non-surgical spermatogonial transplantation directly into the testes of adult fish. The results revealed that, approximately two months following transplantation, testes of recipient sexually mature tilapia are capable of generating functional fertile spermatozoa and generate progeny with the donor genotype. Within a similar timeframe, cryopreserved and thawed tilapia spermatogonia formed spermatozoa in recipient testes. A number of important applications can be derived from these investigations. In combination with in vitro cultures, genetic modification and cryopreservation of spermatogonial stem cells (also performed in the present study), this technique is expected to improve fish bioengineering, such as fish production using transgenic spermatogonia. It may also have significance in the propagation of endangered fish species and may facilitate the seed production of commercially valuable fish species. Also, to extend these possibilities in this group of vertebrates, it has been demonstrated that adult spermatogonia are able to generate both spermatozoa and oocytes [5].

The sequential analysis over different periods of time following syngeneic transplantation revealed that, after colonization, donor spermatogonia proliferate and result in the production of functional spermatozoa in the adult recipient gonad within a very short period (∼9 weeks) post-transplantation. These donor-derived gametes produced normal progeny through natural fertilization and their genotypes were detected in 6.3% of the individuals from the F1 generation. Considering the very high similarities between these two closely related tilapias strains investigated (chitralada and red tilapia) [17], this result is in full agreement with the percentage data inferred from the literature related to fish studies in this field that ranged from 3.8 to 5.2% [5], [7], [25], [26]. Therefore, from the results observed in the literature and in our study we could conclude that our success rate was quite good, mainly when one considers that some or even many of the other 30 larvae evaluated could eventually present another donor marker if other specific loci were investigated.

The very short period necessary for fertile spermatozoa to be formed in tilapia after germ cells transplantation contrasts with the technique developed for salmonids, where it takes more than a year to produce fertile spermatozoa following the transplantation of germ cells in newly hatched larvae [4], [6]. Moreover, as colonization efficiency in this approach is affected by recipient age, donor cells must be transferred during a very narrow timeframe in recipient embryonic development [27]. Overcoming all these limitations, the methods described in the present study have the potential to be widely applicable to many fish species.

Other studies have demonstrated that, when transplanted by microinjection into recipient fish blastulas, single primordial germ cells from different fish families [28] and also blastomers [29], [30], [31] are incorporated, giving rise to germ line chimeras that produce functional sperm with donor genetic characteristics. However, donor and recipient blastulas need to be in the same stage of development, which can be difficult to determine. While feasible, these methods require the long-term rearing of recipient animals. In contrast, the use of sexually mature recipient fish, as established in mammals [32] and demonstrated in the present study, can facilitate and considerably shorten the time needed to obtain donor-derived gametes and offspring. In our study, the necessary time for fertile gamete formation was about two months following transplantation. It is known that, in Nile tilapia, the combined duration of meiotic and spermiogenic phases (preleptotene/leptotene up to spermatozoa), is approximately 10–11 days [33]. A delay in the development of donor-derived spermatogenesis after transplantation had been reported previously [34], [35]. The re-establishment of exogenous spermatogenesis in recipient testis is not immediate. In mammals [34], [35], [36], [37], it was shown that transplanted spermatogonial stem cells require at least one week to establish themselves in the seminiferous tubule niche (colonization), replace their population (proliferation) and later differentiate to form cells committed to spermatogenesis. Usually, spermatogonial cells give rise to spermatocytes after a fixed number of mitotic divisions, depending on the species considered [38], [39]. Eight spermatogonial generations are observed in Nile tilapia [33], therefore, the yet undetermined time spent during the mitotic/spermatogonial phase of spermatogenesis, must be also considered.

To our knowledge, our study is the first to cryopreserve and after freezing/thawing to functionally evaluate adult spermatogonia in fish. Apparently, in the present investigation it took a little bit longer to form spermatozoa from frozen/thawed spermatogonia. Despite of the short delay in the development of donor-derived spermatogenesis, we demonstrate that tilapia spermatogonial stem cells can be successfully cryopreserved and are able to maintain their functionality after cryopreservation. A protocol for cryopreservation of rainbow trout (*Oncorhynchus mykiss*) PGCs and the generation of viable gametes derived from these cryopreserved progenitor cells were already been reported [26]. However, unlike the technique described here, as the thawed PGCs were transplanted into the coelomic cavities of trout hatchlings, they were able to differentiate into gametes only when recipients reach sexual maturity, i.e. approximately in two years. Cryopreservation of the male germ line effectively establishes the potential to preserve threatened fish species genome. Considering the endangered status of many species of cichlids [40], [41], the cryopreservation and interspecific transplantation of their spermatogonia into the testis of adult tilapia could make it possible to regenerate the target species, even after this species is eventually extincted. Moreover, spermatogonial cryopreservation following transplantation also has valuable implications in the production of commercially valuable strains or species of fish.

Extending the range of questions addressing the fish spermatogenesis and spermatogonial stem cell biology, the methodology applied in this study could be also feasible for interspecific/xenogeneic transplantation. In this context, preliminary investigation in our laboratory has indicated that germ cells from *Cichla monoculus*, an Amazon cichlid, were able to colonize and form spermatogenic cysts in the tilapia seminiferous epithelium, suggesting that the testicular microenvironment of adult tilapia may provides the necessary conditions for the development of spermatogenesis, at least from different species of cichlid fish. In this way, besides facilitating seed production, lifecycle periods of a given fish species could be significantly shortened if, for instance, the surrogate parent species present a shorter period until reaching puberty. Moreover, commercially valuable species that require demanding labor and high production cost, or even those that need more rearing space or have seasonal reproduction, can potentially have their gamete production facilitated and increased using the appropriate recipient fish species [42]. Taking into account all the positive attributes mentioned in the present investigation, it seems that the tilapia is an excellent recipient model.

**Figure 5:**
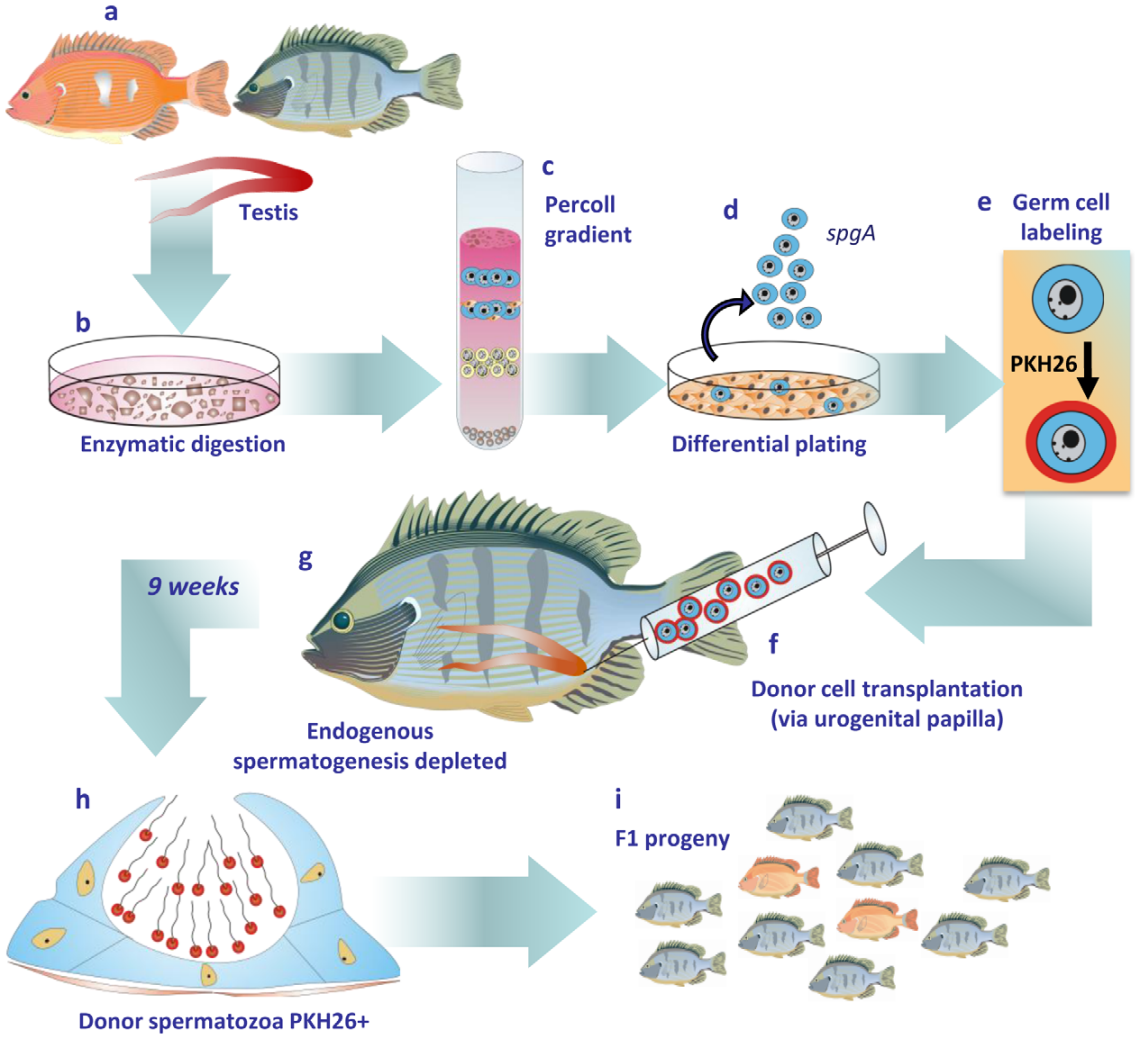
Illustration of the main steps utilized for germ cell transplantation in sexually mature tilapia (*O. niloticus*). Donor testes were removed from tilapia (a) and these testes were incubated in a dissociating solution containing the enzymes trypsin, collagenase and DNAse (b). The obtained cell suspension was submitted to percoll density gradient centrifugation (c) in order to enrich undifferentiated spermatogonia (*spg*) and for differential plating (d) to remove eventual testicular somatic cells. As there are no genetic markers available for tilapia, PKH26 was used to label the donor spermatogonia (e). The germ cells were transplanted via the common spermatic duct located in the urogenital papillae (f) into recipient tilapia that had their endogenous spermatogenesis previously depleted with busulfan (g). Recipient tilapia were sacrificed at different time intervals post-transplantation in order to follow the development of PKH26-labeled germ cells. Transplanted germ cells were able to colonize the recipient testis and gave rise to fertile spermatozoa at approximately 9 weeks post-transplantation (h) and these spermatozoa resulted in offspring with donor genetic characteristics (i).

The procedures reported in the present investigation on spermatogonial transplantation in adult Nile tilapia are illustrated in Fig. 5. Based on our results, a new, effective, fast non-surgical germ cell transplantation technique was developed, allowing a feasible method for producing fertile fish sperm. In association with germ cell cryopreservation, this new technique would probably be suitable for studies related to biotechnology in aquaculture, also providing possibilities for transgenesis, the preservation of endangered species and genetic stocks of valuable fish species. Among several other opportunities, an easy system was also developed for possibly evaluating spermatogonial stem cell biology and spermatogenesis in fishes.

## Supporting Information

Figure S1 Confocal microscopy analysis of tilapia testis five weeks after spermatogonial transplantation. Suggesting that transplanted spermatogonia can self-renew and/or stay longer in the testes, isolated PKH26-labeled spermatogonia (in red; arrow), surrounded by somatic cells in green, are still observed in the recipient seminiferous epithelium several weeks after transplantation. The insert shows donor cell (spgA) at a higher magnification. Green fluorescence represents labeling of actin filaments. TA: tunica albuginea. Scale bar = 10µm.
(1.38 MB TIF)

Figure S2 Illustrative figure from experiments related to the crossing of red tilapia (O. niloticus) with Chitralada tilapia (O. niloticus). Note that most fish present typical Chitralada tilapia skin pigmentation (black arrowhead), whereas, in approximately 50% of the fishes, the skin pigmentation is similar to the red tilapia (∼1/4; gray arrowhead) or is spotted (∼1/4; blue arrowhead). Scale bar: 3 cm.
(1.29 MB TIF)

Table S1 Donor-derived spermatogenesis in the recipient tilapia following syngenic transplantation.
(0.05 MB DOC)

Table S2 Donor-derived spermatogenesis in recipient tilapia following transplantation of cryopreserved spermatogonia.
(0.04 MB DOC)
