# Supplementary figures and images for: A New and Fast Technique to Generate Offspring after Germ Cells Transplantation in Adult Fish: The Nile Tilapia (Oreochromis niloticus) Model
Source: PLoS One. 2010 May 20;5(5):e10740. doi: 10.1371/journal.pone.0010740 (PMC2873995; doi:10.1371/journal.pone.0010740)

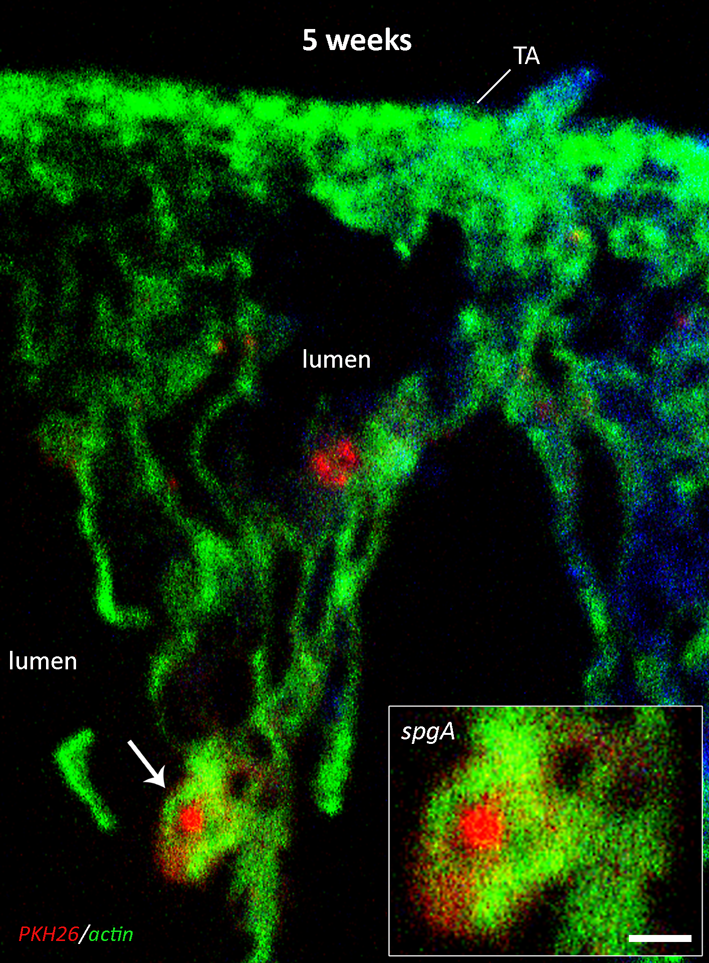

Supplement: Figure S1 — Confocal microscopy analysis of tilapia testis five weeks after spermatogonial transplantation. Suggesting that transplanted spermatogonia can self-renew and/or stay longer in the testes, isolated PKH26-labeled spermatogonia (in red; arrow), surrounded by somatic cells in green, are still observed in the recipient seminiferous epithelium several weeks after transplantation. The insert shows donor cell (spgA) at a higher magnification. Green fluorescence represents labeling of actin filaments. TA: tunica albuginea. Scale bar = 10µm. (1.38 MB TIF) [file pone.0010740.s001.tif]

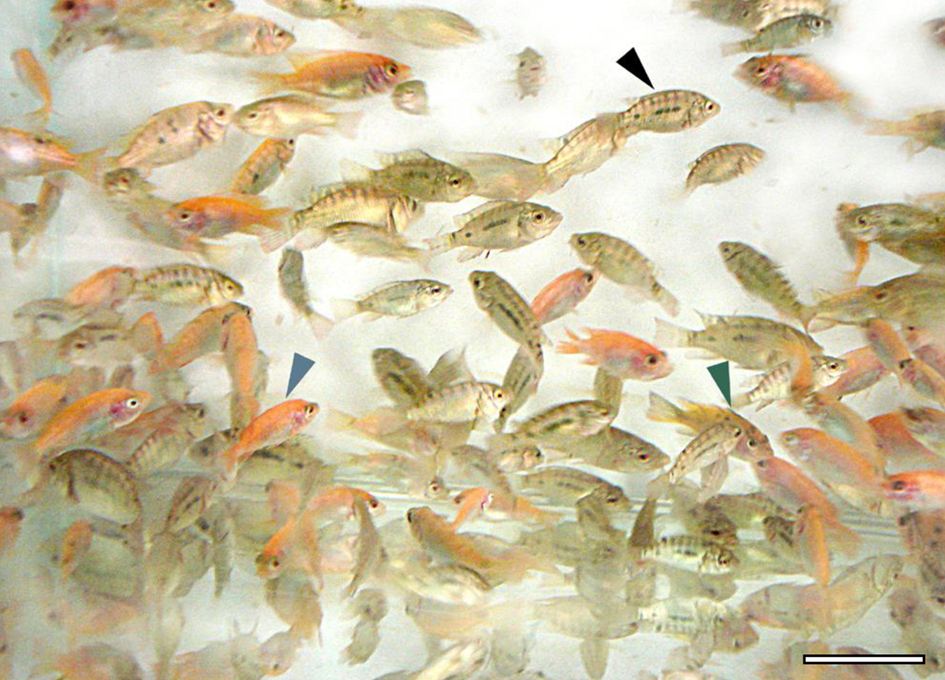

Supplement: Figure S2 — Illustrative figure from experiments related to the crossing of red tilapia (O. niloticus) with Chitralada tilapia (O. niloticus). Note that most fish present typical Chitralada tilapia skin pigmentation (black arrowhead), whereas, in approximately 50% of the fishes, the skin pigmentation is similar to the red tilapia (∼1/4; gray arrowhead) or is spotted (∼1/4; blue arrowhead). Scale bar: 3 cm. (1.29 MB TIF) [file pone.0010740.s002.tif]
