# Supplementary material for: A New and Fast Technique to Generate Offspring after Germ Cells Transplantation in Adult Fish: The Nile Tilapia (Oreochromis niloticus) Model
Source: PLoS One. 2010 May 20;5(5):e10740. doi: 10.1371/journal.pone.0010740 (PMC2873995; doi:10.1371/journal.pone.0010740)
Supplement: Table S1 — Donor-derived spermatogenesis in the recipient tilapia following syngenic transplantation. (0.05 MB DOC) [file pone.0010740.s003.doc]

| **Table S1 -** Donor-derived spermatogenesis in the recipient tilapia following syngenic transplantation. | | | |
| --- | --- | --- | --- |
| **Post-transplantation period** | **No. of recipient fish analyzed** | **Gonads with donor-derived cells (%)** | **Gonads with donor-derived spermatocysts (%)** |
| 1 hour | 4 | 100.0 | NA |
| 14 hour | 2 | 100.0 | NA |
| 2 weeks | 4 | 100.0 | 75.0 |
| 3 weeks | 3 | 33.3 | 33.3 |
| 4 weeks | 5 | 60.0 | 40.0 |
| 5 weeks | 5 | 80.0 | 60.0 |
| 6 weeks | 6 | 66.6 | 66.6 |
| 7 weeks | 5 | 100.0 | 80.0 |
| 8 weeks | 3 | 100.0 | 100.0 |
| 9 weeks | 3 | 100.0 | 100.0 |

| **Table S1 -** Donor-derived spermatogenesis in the recipient tilapia following syngenic transplantation. | | | |
| --- | --- | --- | --- |
| **Post-transplantation period** | **No. of recipient fish analyzed** | **Gonads with donor-derived cells (%)** | **Gonads with donor-derived spermatocysts (%)** |
| 1 hour | 4 | 100.0 | NA |
| 14 hour | 2 | 100.0 | NA |
| 2 weeks | 4 | 100.0 | 75.0 |
| 3 weeks | 3 | 33.3 | 33.3 |
| 4 weeks | 5 | 60.0 | 40.0 |
| 5 weeks | 5 | 80.0 | 60.0 |
| 6 weeks | 6 | 66.6 | 66.6 |
| 7 weeks | 5 | 100.0 | 80.0 |
| 8 weeks | 3 | 100.0 | 100.0 |
| 9 weeks | 3 | 100.0 | 100.0 |
