# Supplementary material for: A New and Fast Technique to Generate Offspring after Germ Cells Transplantation in Adult Fish: The Nile Tilapia (Oreochromis niloticus) Model
Source: PLoS One. 2010 May 20;5(5):e10740. doi: 10.1371/journal.pone.0010740 (PMC2873995; doi:10.1371/journal.pone.0010740)
Supplement: Table S2 — Donor-derived spermatogenesis in recipient tilapia following transplantation of cryopreserved spermatogonia. (0.04 MB DOC) [file pone.0010740.s004.doc]

| **Table S2 -** Donor-derived spermatogenesis in recipient tilapia following transplantation of cryopreserved spermatogonia. | | | |
| --- | --- | --- | --- |
| **Post-transplantation period** | **Individual Recipient** | **Donor-derived cells (Colonization)** | **Donor-derived spermatocysts** |
| 4 week | 1 | Present | Absent |
| 8 weeks | 2 | Present | Absent |
| 8 weeks | 3 | Present | Present |
| 9 weeks | 4 | Absent | Absent |
| 10 weeks | 5 | Present | Present |
| 10 weeks | 6 | Present | Present |
| 11 weeks | 7 | Present | Present |
| 11 weeks | 8 | Present | Present |
